# Editorial: Mitochondrial dysfunction in cellular and molecular mechanisms of brain aging

**DOI:** 10.3389/fnagi.2026.1866752

**Published:** 2026-05-25

**Authors:** Ying Li, Aswathy Peethambaran Mallika, Meghraj Singh Baghel, Bhupendra V. Shravage, Pei Shang

**Affiliations:** 1 Department of Neurology, Nanfang Hospital, Southern Medical University, Guangzhou, Guangdong, China; 2 Department of Pathology, Johns Hopkins School of Medicine, Baltimore, MD, United States; 3 Developmental Biology Group, Agharkar Research Institute, Pune, India; 4 Department of Biotechnology, Savitribai Phule Pune University, Pune, India; 5 Department of Neurology, Mayo Clinic, Rochester, MN, United States

**Keywords:** aging, Alzheimer's disease, mitochondria, oxidative phosphorylation (OXPHOS), Parkinson's disease

Brain aging underlies many neurological disorders, including Alzheimer's disease (AD), Parkinson's disease (PD), primary age-related tauopathy (PART), limbic-predominant age-related TDP-43 encephalopathy (LATE), Lewy body dementia (LBD), and chronic post-traumatic brain dysfunction. Mitochondria play a significant role in energy metabolism and signal transduction. Abnormal mitochondrial energy metabolism and redox imbalance are the hallmark changes during brain aging. Mitochondrial DNA (mtDNA) is more susceptible to oxidative damage than nuclear DNA. Under conditions of cellular stress, such as excessive oxidative stress or immune activation, mtDNA is more prone to damage and release into the cytosol, which may trigger inflammatory and innate immune responses (Wen et al., 2026). Mitochondrial dysfunction promotes protein aggregation, immune activation, and inflammatory responses, creating a vicious cycle of aging. Several studies have now found that mitochondrial dysfunction is positively correlated with aging-associated diseases. This Research Topic includes six studies on the impact of mitochondrial dysfunction on brain aging, which are demonstrated from multiple perspectives such as intestinal microecology, immune cell activation, pathological protein toxicity, and metabolic redox regulation. These studies highlight mitochondrial dysfunction as a central contributor to brain aging and suggest potential avenues for neuroprotective interventions.

Fecal microbiota transplantation (FMT) regulates PD through the microbiota-gut-brain axis. The gut microbiota of PD patients often shows an increase in pathogenic bacteria and a decrease in beneficial bacteria, and this imbalance of the microbiota can exacerbate neuroinflammation through neural, immune, endocrine and other pathways. The advantage of FMT lies in its ability to restore the balance of the microbiota, thereby improving some symptoms of PD patients. By integrating animal experiments and clinical data, the study also found that the FMT via the colorectal pathway is more comprehensive in efficacy, and oral capsules are more acceptable. The microbiota-gut-brain regulation approach may offer new targeted treatments for PD patients. In addition, gut microbiota profiles (the target of FMT) could serve as potential early biological markers for PD diagnosis (Wang et al.).

Traumatic brain injury (TBI) is associated with abnormalities in immune function and mitochondrial homeostasis. The study constructed single-cell atlases of multiple tissues at multiple time points through single-cell RNA sequencing, and conducted pseudo-temporal analysis, ligand-receptor (LR) analysis, and *in vitro* lipopolysaccharide (LPS) stimulation verification. At the two time points of 24 h after injury and the 7th day, the study revealed that TBI induces cross-tissue, time-specific myeloid cell remodeling and the continuous increase of cortical activated microglia. These immune cells are overactivated and release a large amount of pro-inflammatory factors, thereby promoting sustained neuroinflammation and mitochondrial oxidative stress, which may accelerate brain aging. This finding may offer new therapeutic options, explain the molecular mechanism of age-related TBI prognosis differences, and provide a theoretical basis for targeted immune therapy for TBI (Sun et al.).

In order to better understand the dynamic characteristics of insoluble aggregation proteins in mitochondria and to distinguish the pathological mechanisms of AD driven by amyloid beta (Aβ)/Tau from normal aging, the study used Caenorhabditis elegans as a model. By analyzing mitochondrial Sarkosyl-insoluble proteins via quantitative proteomics (LC-MS/MS) and detecting post-translational modifications (PTMs), the study confirmed that Aβ and Tau induce mitochondrial protein aggregation. The normal aging model is characterized by progressive protein aggregation; the number of aggregated proteins in the Aβ model is far greater than that in the other models, leading to extensive proteostasis disruption and bioenergetic stress; the Tau model shows early-onset and specific transport system disorders, and the peak time of protein aggregation in the Tau model and the Aβ model is earlier than that in the normal aging model. Aβ triggers energy metabolism disorders through early oxidative stress and abnormal aggregation of metabolic enzymes, while Tau exacerbates protein homeostasis imbalance by disrupting the cellular transport system and nuclear-cytoplasmic transport. The pathways of action are different, but both ultimately lead to mitochondrial dysfunction. This discovery clarifies the differences in mitochondrial aggregation protein characteristics between aging and AD, providing molecular markers for distinguishing physiological aging from pathological aging (Pahal et al.).

According to the oxidative-reductive theory of aging, the imbalance of key redox pairs within cells (including [NADPH]/[NADP^+^], [NAD^+^]/[NADH], etc.) is one of the important causes of aging. The review within this Research Topic integrates the detection of metabolite ratios and synthesizes data from different animal models, finding that brain mitochondrial redox ratios share certain cross-species commonalities: mitochondrial [NAD^+^]/[NADH] is universally shifted toward an oxidized state with aging, whereas cytoplasmic redox ratio changes exhibit species- and strain-specific characteristics. Through interventions such as dietary restriction and intermittent fasting, pathways such as fatty acid β-oxidation and mitochondrial shuttle systems can be activated, inducing cyclic reduced-state redox shift, reversing mitochondrial oxidative imbalance, and stabilizing the cytoplasmic redox state, ultimately achieving the effect of delaying brain aging (Jamerson et al.).

This Research Topic also includes rare genetic diseases related to mitochondria function. *MSTO1* is a nuclear gene encoding a mitochondrial membrane protein involved in maintaining mitochondrial network dynamics, including fission and fusion. Pathogenic variants in *MSTO1* disrupt mitochondrial function and cause an autosomal recessive disorder characterized by myopathy and ataxia. However, the symptoms, genetic links, and long-term progression of *MSTO1*-related disorders are still not well understood. In this study, Wu et al. report a patient with adult-onset cerebellar ataxia who carries two novel *MSTO1*variants (c.756A>G and c.1339G>A) both initially classified as variants of uncertain significance. The expression of MSTO1 protein was significantly reduced, and the mitochondrial respiratory capacity and potential were decreased. These findings expand the phenotypic spectrum of MSTO1-related mitochondrial disease to include adult-onset, mild cerebellar ataxia type. Their findings link these variants to mitochondrial dysfunction and expand the disease spectrum to include a milder, adult-onset form of cerebellar ataxia. They also highlight the importance of combining clinical and functional analyses to assess variant pathogenicity and understand disease variability (Wu et al.).

From a therapeutic perspective for AD, Pinky et al. investigated the cognitive-enhancing and anti-amnesic effects of propranolol, a β-adrenergic antagonist commonly used to treat high blood pressure, irregular heart rhythms, anxiety and migraines. Propranolol has also been suggested to benefit individuals with behavioral and psychological symptoms of dementia. This study shows that in a scopolamine-induced rat model, propranolol improved memory, recognition, and anxiety-like behavior. These effects were associated with enhanced synaptic plasticity signaling, improved mitochondrial function, reduced oxidative stress, and decreased inflammation. Overall, the study discusses the potential therapeutic value of propranolol for AD-related cognitive impairment (Pinky et al.).

This Research Topic's six studies, from different perspectives such as the regulation of the microbiota-gut-brain axis, post-traumatic immune inflammation, mitochondrial protein homeostasis, and redox metabolism, jointly confirmed the important viewpoint that mitochondrial dysfunction represents a central node in the cellular and molecular mechanisms of brain aging. It is not only an important product of the aging process but also a key driving force for the continuous progression of brain aging. These studies have demonstrated the key mechanisms and innovations. However, current research still has limitations such as limited translatability, insufficiently in-depth mechanistic insights, and unclear cell/tissue specificity. Nevertheless, these findings not only deepen our understanding of the essence of brain aging but also provide a clear direction for future mitochondrial-targeted interventions, potentially contributing to an important breakthrough in delaying brain aging and promoting healthy brain aging.
